# Estimates of influenza and respiratory syncytial virus incidences with fraction modeling approach in Baguio City, the Philippines, 2012‐2014

**DOI:** 10.1111/irv.12453

**Published:** 2017-05-03

**Authors:** Taro Kamigaki, Portia P. Aldey, Edelwisa S. Mercado, Alvin G. Tan, Jenaline B. Javier, Socorro P. Lupisan, Hitoshi Oshitani, Veronica L. Tallo

**Affiliations:** ^1^ Department of VirologyTohoku University Graduate School of MedicineSendaiJapan; ^2^ Department of Epidemiology and BiostatisticsDepartment of HealthResearch Institute for Tropical MedicineManilaPhilippines; ^3^ Molecular Biology LaboratoryDepartment of HealthResearch Institute for Tropical MedicineManilaPhilippines; ^4^ Department of HealthResearch Institute for Tropical MedicineManilaPhilippines

**Keywords:** health care‐seeking behavior, incidence, influenza, respiratory syncytial virus

## Abstract

**Background:**

Estimation of the incidences of influenza and respiratory syncytial virus (RSV) infection is important for disease control. Previous estimate in the city showed a substantial burden of influenza in both outpatients and inpatients while it did not account for individuals who do not seek medical attention nor RSV.

**Patients/Methods:**

A total of 17 674 influenza‐like illness (ILI) and 13 242 severe acute respiratory illness (SARI) cases were recruited, and samples were collected from 6267 and 2962 of ILI and SARI cases, respectively. RT‐PCR assays were performed to detect influenza and RSV in the samples. A health‐seeking behavior survey was conducted from February 2014 to April 2014 to estimate the fraction of infected individuals who did not seek medical attention between rainy and dry season.

**Results:**

Average influenza and RSV incidence rates in outpatients were 1.6 and 1.4 per 1000 individuals, respectively, and the highest incidence rate for both viruses was found in the of 6‐23 month age group. Average influenza and RSV hospitalization incidence rates were 1.7 and 1.9 per 1000 individuals, respectively. Further, we estimated that the incidence rates of influenza and RSV in individuals who did not seek medical attention were threefold and 1.6‐fold those in the medically attended population.

**Conclusions:**

Respiratory syncytial virus and influenza pose a substantial disease burden, particularly in hospitalized cases. The implementation of either a community‐based approach or an enhanced surveillance system in combination with a community survey will allow a better understanding of the disease burdens of RSV and influenza in the Philippines.

## 1 Introduction

Recently, general understanding of the influenza disease burden has improved globally.^1^, ^2^ This information is essential for policymakers to appropriately allocate resources to implement control measures such as vaccination. In the Philippines, an enhanced surveillance system to monitor influenza‐like illness (ILI) and severe acute respiratory infection (SARI) began in 2009. Substantial influenza incidence including the 2009 H1N1 pandemic was estimated for outpatients and inpatients during the period of 2009‐2011.^3^ In spite of this work, there remains an important gap in estimating influenza incidence in the Philippines, largely owing to the presence of an influenza‐infected population that does not seek medical attention during their illness.

Respiratory syncytial virus (RSV) causes acute respiratory illness, particularly in young children.^4^ Estimates of RSV incidence are highly variable across study populations,^5^ and the incidence of this disease is sparsely reported, particularly in developing countries in tropical regions.^6^, ^7^ Currently some vaccines for RSV are in the trial phase, and RSV incidence rate is one of the primary endpoints being used to evaluate the effectiveness of these vaccines.^5^ Additionally, estimates of the RSV incidence rate provide useful information for prioritizing the implementation of public health services within a country.

This study aimed to estimate the incidence rates of influenza and RSV in Baguio City as well as to compare estimates of non‐medically attended ILI episodes with health‐seeking behavior data.

## 2 Materials and Methods

### 2.1 Study sites

Baguio City is the regional center of the Cordillera Administrative Region, which is located in the northern part of Luzon Island, and has a population of 315 000. In the city, there are 16 public health centers (PHCs) providing a variety of primary medical services including immunization and morbidity consultations to inhabitants in the catchment areas. In addition to the PHCs, five hospitals and private clinics are major facilities to provide medical services.

### 2.2 ILI and SARI surveillance

The details of ILI and SARI surveillance in the Philippines have been previously documented.^3^ Briefly, an ILI case is defined as a patient who developed a sudden onset of a fever (>38°C) with a cough or sore throat. A SARI case is defined as a patient who developed shortness of breath or difficulty in breathing that required hospital admission. Children who were diagnosed with cases of pneumonia or severe pneumonia according to the integrated management of childhood illness guidelines^8^ were also enrolled in our study. ILI and SARI surveillance are operated in all PHCs and hospitals in the city. A structured questionnaire was used, and naso‐ or oropharyngeal swabs were collected by field nurses on each surveillance day. The surveillance day was one of the two morbidity consultation days in PHCs. Field nurses visited to hospital once a week to collect specimens from SARI cases if the interval days from onset did not excess to 5 days. The weekly numbers of ILI and SARI cases who visited on days other than these surveillance days were then counted. All collected specimens were tested for influenza A and its subtypes (hereafter, A(H1)pdm09 or A(H3)) by performing RT‐PCR assays^9^ or tested for influenza B and RSV by performing conventional RT‐PCR assays.^10^ The study period was between 1 January 2012 and 31 December 2014.

### 2.3 Health‐seeking behavior survey

Each PHC has a catchment area which consists of several barangays (local administrative division) for their health services. All PHCs were categorized into three groups according to ILI consultation activity level (low, moderate, or high) and into two groups by their shortest distance from city hall (suburb or city center). The consultation activity level was defined by the average number of annual ILI consultations reported in 2012 and 2013. All PHCs were allocated in the above 2×3 matrix (2‐3 PHCs in each cell), and a PHC was each randomly selected from the cell (totally 6 PHCs). Two or three barangays were then randomly selected for each PHC covering area. The total sample size was calculated as 2800 based on the assumption that 50% of the population^11^ would have sought medical consultation with a precision of 5%. A design effect of two was taken into account owing to the clustering of cases by barangay and household. The sample design in the survey was a self‐weighting. The number of study population was proportionally allocated by population size in each barangay and also by the age structure of population in the city. The survey was conducted between February 2014 and April 2014. All interviews were conducted with a person of targeted age group by trained interviewers, and a field coordinator supervised their activities. A standardized questionnaire was used to obtain respondents' demographic and socioeconomic information, household structure, and an attitude as well as practice to seek medical consultation after developing ILI‐ and SARI‐like symptoms (Supplement information). Symptoms in case definitions of both ILI and SARI were explained during the interview. We asked all participants about experiences of both ILI and SARI episodes 2 months prior to the survey. We then estimated frequencies of different health‐seeking behavior in ILI and SARI episodes. The survey was conducted during dry season in the city.

### 2.4 Statistical analysis

The virus‐positivity rate was calculated as the proportion of the number of positive samples to the total number of tested samples. The population was projected based on the 2010 census data. We estimated the influenza incidence rate by each age group, as expressed per 1000 individuals, by dividing the number of patients with ILI who presented at the health facilities and whose samples tested positive for influenza by the census population. Then, we multiplied the result with the inverse of the proportion of the number of samples obtained to the total ILI count. A similar procedure was used for estimating RSV‐ and influenza‐associated SARI incidence rates. We estimated the overall influenza incidence rate based on cases of A(H1)pdm09, A(H3), and influenza B. A 95% confidence interval (CI) was estimated using simple exact binomial CIs.

A Bayesian regression model was constructed to estimate the number of ILI cases who sought care at health facilities other than PHCs from the number of cases who visited PHCs:

$$y_{i,j,k}=c+b_{1}X_{i,j,k}+b_{2}C_{i,j}+b_{3}S_{i,j}$$

where *Y*_*i,j,k*_ and *X*_*i,j,k*_ are the number of ILI cases during the rainy season and dry seasons, respectively, at age group *i*, category *j*, and distance *k*. *C*_*i,j*_ and *S*_*i,j*_ are the rate of PHC consultation activity and the rate of intent to seek consultations during the rainy season (June to December), respectively. The fraction of non‐medically attended to medically attended cases was then applied to obtain the total number of ILI consultations in each PHC area. All analyses were performed in R version 3.1.0,^12^ and rstan version 2.8.0 was used to construct the model.^13^

### 2.5 Ethical issues

Written informed consent was obtained prior to participation, primarily from the respondents or their caretakers if interviewees are children. Approval for the study design and protocol was obtained from the Ethical Review Committee of the Tohoku University Graduate School of Medicine (ID 2013‐1‐410) and the Research Institute of Tropical Medicine Institutional Review Board.

## 3 Results

### 3.1 Virus‐positive samples among ILI and SARI cases

**Figure 1:**
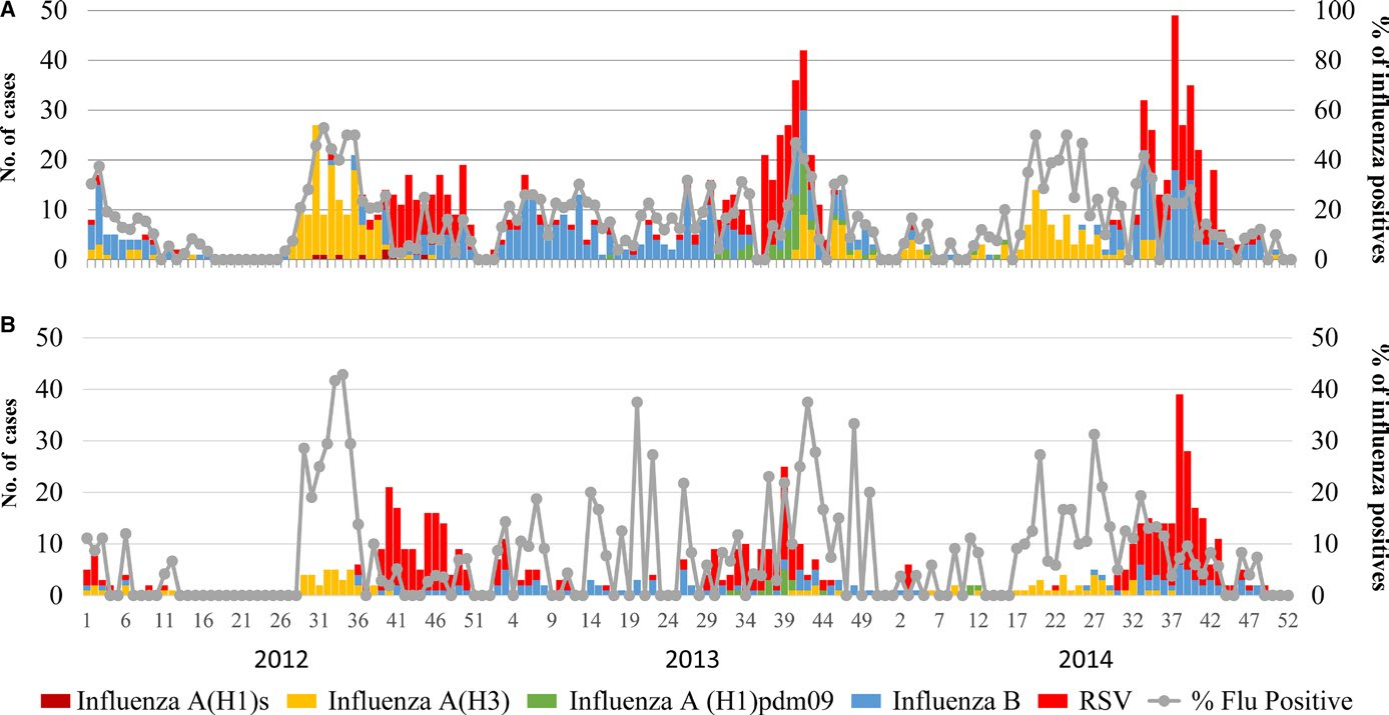
(A, B) Weekly number of influenza subtype and respiratory syncytial virus (RSV) positives and the percentage of influenza positives, 2012‐2014, Baguio City, the Philippines

In total, 17 674 ILI and 13 242 SARI cases were recruited throughout the study period. The number of both ILI and SARI cases was not different between rainy and dry seasons (*P*=.99 respectively). Of those, samples were collected from 6267 ILI cases (35.5%) and 2962 SARI cases (22.4%). When comparing the ages of the ILI and SARI patients, we found that children accounted for the highest number of cases with ILI, while the highest number of SARI cases was in adults over 50 years of age (Table 1). Fifteen of the SARI cases were fatal; however, except for one case each with influenza B and RSV, who both returned home against medical advice, all of these patients tested negative for the targeted viruses. For the ILI cases, influenza‐positive samples were detected during 84.6% of the study weeks, while RSV‐positive samples were only detected during 53.9% of the study weeks (Figure 1).

**Table 1:** The numbers of total case and cases with sampling among ILI and SARI, 2012‐2014

Category	Year	Number	<6 mo	6‐23 mo	2‐4 y	5‐14 y	15‐49 y	≥50 y
ILI	2012	Total	276	1455	1527	1230	488	82
		Sample taken	79	480	487	321	98	12
	2013	Total	295	1533	1614	1660	631	105
		Sample taken	93	490	499	433	139	22
	2014	Total	245	1421	1355	1433	493	90
		Sample taken	69	424	402	364	103	11
SARI	2012	Total	354	762	577	380	437	1078
		Sample taken	133	284	172	71	76	160
	2013	Total	511	964	581	415	525	1296
		Sample taken	144	305	137	74	61	150
	2014	Total	791	1348	696	478	552	1497
		Sample taken	237	476	165	123	69	125

ILI, influenza‐like illness; SARI, severe acute respiratory illness.

The virus with the highest number of positives varied across the study years (Table 2). For influenza A, A(H3) virus predominantly circulated in 2012 and 2014 whereas A(H1)pdm09 and A(H3) cocirculated in 2013. The numbers of influenza B‐ and RSV‐positive samples were the highest among the age groups of 5‐14 years and 6‐23 months old, respectively. The highest numbers of A(H1)pdm09 and A(H3) cases were both found among the age group of 2‐4 years. Of the ILI and SARI cases whose samples were collected, 2.4% and 9.4%, respectively, had received an influenza vaccination within a year prior to their illness. Within these subsets, five A(H3) and 11 influenza B cases were detected among the ILI cases, while 11 A(H3), three A(H1)pdm09, and 20 influenza B cases were detected among the SARI cases. A(H3)‐ and influenza B‐positive cases in the SARI group were found most commonly in the <2 year age group.

**Table 2:** A, B The number and positivity proportion of influenza and RSV positives by age groups in ILI (A) and SARI (B), 2012‐2014

(A)								
Year	Viruses	Total	<6 mo	6‐ 23 mo	2‐4 y	5‐14 y	15‐49 y	≥50 y
2012	A(H1)pdm09	0	0(0)	0(0)	0(0)	0(0)	0(0)	0(0)
	A (H3N2)	150	4(5.1)	32(6.7)	68(14.0)	34(10.6)	12(12.2)	0(0)
	B	87	2(2.5)	20(4.2)	29(6.0)	32(10.0)	4(4.1)	0(0)
	RSV	130	7(8.9)	64(13.3)	43(8.8)	15(4.7)	0(0)	1(8.3)
2013	A(H1)pdm09	50	0(0)	9(1.8)	19(3.8)	19(4.4)	3(2.2)	0(0)
	A (H3N2)	36	1(1.1)	5(1.0)	15(3.0)	12(2.8)	3(2.2)	0(0)
	B	241	10(10.8)	41(8.4)	59(11.8)	104(24.0)	25(18.0)	2(9.1)
	RSV	173	9(9.7)	75(15.3)	59(11.8)	23(5.3)	7(5.0)	0(0)
2014	A(H1)pdm09	4	0(0)	1(0.2)	1(0.2)	1(0.3)	0(0)	1(9.1)
	A (H3N2)	99	4(5.8)	15(3.5)	32(8.0)	29(8.0)	16(15.5)	3(27.3)
	B	146	0(0)	23(5.4)	27(6.7)	78(21.4)	15(14.6)	3(27.3)
	RSV	151	7(10.1)	69(16.3)	57(14.2)	16(4.4)	1(1.0)	1(9.1)

(B)								
2012	A(H1)pdm09	0	0(0)	0(0)	0(0)	0(0)	0(0)	0(0)
	A (H3N2)	44	2(1.5)	8(2.8)	12(7)	7(9.9)	5(6.6)	10(6.3)
	B	20	2(1.5)	8(2.8)	5(2.9)	2(2.8)	1(1.3)	2(1.3)
	RSV	148	36(27.1)	66(23.2)	27(15.7)	5(7)	5(6.6)	9(5.6)
2013	A(H1)pdm09	17	0(0)	4(1.3)	4(2.9)	3(4.1)	1(1.6)	5(3.3)
	A (H3N2)	7	0(0)	2(0.7)	1(0.7)	2(2.7)	0(0)	2(1.3)
	B	74	4(2.8)	26(8.5)	9(6.6)	15(20.3)	11(18)	9(6)
	RSV	124	36(25)	61(20)	14(10.2)	4(5.4)	4(6.6)	5(3.3)
2014	A(H1)pdm09	7	1(0.4)	2(0.4)	1(0.6)	1(0.8)	1(1.4)	1(0.8)
	A (H3N2)	39	2(0.8)	12(2.5)	3(1.8)	6(4.9)	5(7.2)	11(8.8)
	B	78	11(4.6)	27(5.7)	9(5.5)	18(14.6)	4(5.8)	9(7.2)
	RSV	179	49(20.7)	93(19.5)	27(16.4)	3(2.4)	2(2.9)	5(4)

ILI, influenza‐like illness; SARI, severe acute respiratory illness; A(H1)pdm09, influenza A(H1N1)pdm09; A(H3), influenza A(H3N2); RSV, respiratory syncytial virus.

We estimated the incidence rates of influenza and RSV among the ILI cases by study year and age group (Table 3). Overall, the average influenza and RSV incidence rates were 1.6 and 1.4 per 1000 individuals, respectively, and the highest incidence rate for both viruses was found in the of 6‐23 month age group. Interestingly, the ratio of incidence rates in the of 2‐4 year age group to that in the 6‐23 month age group was significantly lower for RSV than that for influenza (0.38 and 0.62, respectively). For both RSV and influenza, children aged <6 months generally had a lower incidence rate than either of those two groups. This trend is more distinct for the RSV incidence rate, while the influenza incidence rates in these three groups were more varied among study years and virus types.

**Table 3:** Incidence rates per 1000 person years of influenza and RSV‐associated ILI by age groups, 2012‐2014

Year	<6 mo	6‐ 23 mo	2‐4 y	5‐14 y	15‐49 y	≥50 y
2012						
Influenza	3.2(0.7‐9)	7.7(3.3‐15.3)	5.4(1.9‐12.2)	2(0.2‐7.2)	0.1(0‐3.9)	0(0‐3.7)
A(H1)pdm09	0(0‐3.7)	0(0‐3.7)	0(0‐3.7)	0(0‐3.7)	0(0‐3.7)	0(0‐3.7)
A(H3)	4(1.1‐10.3)	12.3(6.4‐21.3)	10.9(5.4‐19.5)	2.4(0.4‐7.8)	0.3(0‐4.2)	0(0‐3.7)
Influenza B	1.8(0.2‐7)	6.3(2.4‐13.5)	4.8(1.5‐11.4)	1.9(0.2‐7.1)	0.1(0‐3.9)	0(0‐3.7)
RSV	6.5(2.5‐13.8)	21.7(13.6‐32.9)	6.8(2.7‐14.1)	1.0(0‐5.4)	0(0‐3.7)	0.1(0‐3.8)
2013						
Influenza	9.3(4.3‐17.4)	14.6(8.1‐24.3)	9.6(4.5‐17.9)	6.1(2.3‐13.2)	0.6(0‐4.9)	0.1(0‐3.9)
A(H1)pdm09	0(0‐3.7)	3.1(0.6‐8.8)	3.54(0.9‐9.6)	1.1(0‐5.7)	0.1(0‐3.9)	0(0‐3.7)
A(H3)	0.8(0‐5.1)	1.6(0.1‐6.5)	2.3(0.3‐7.7)	0.7(0‐5.1)	0.1(0‐3.9)	0(0‐3.7)
Influenza B	9(4.1‐17.1)	14.1(7.7‐23.6)	9.3(4.3‐17.5)	6.1(2.3‐13.2)	0.6(0‐4.9)	0.1(0‐3.9)
RSV	8.9(4‐16.9)	25.6(16.7‐37.6)	11.1(5.6‐19.9)	1.4(0.1‐6.3)	0.1(0‐3.9)	0(0‐3.7)
2014						
Influenza	1.2(0.1‐5.9)	9(4.1‐17)	4.6(1.4‐11.2)	4.4(1.3‐10.9)	0.3(0‐4.3)	0.2(0‐4)
A(H1)pdm09	0(0‐3.7)	0.3(0‐4.3)	0.2(0‐4)	0.1(0‐3.9)	0(0‐3.7)	0.04(0‐3.8)
A(H3)	3.8(1‐9.9)	7.4(3.1‐15)	6.1(2.3‐13.2)	1.8(0.2‐6.9)	0.4(0‐4.5)	0.2(0‐4.2)
Influenza B	0(0‐3.7)	8.1(3.5‐15.9)	4.3(1.3‐10.7)	4.4(1.3‐10.8)	0.3(0‐4.3)	0.2(0‐4)
RSV	6.3(2.4‐13.5)	21.7(13.5‐32.9)	8.7(3.9‐16.7)	0.9(0‐5.4)	0.02(0‐3.7)	0.1(0‐3.8)

ILI, influenza‐like illness; A(H1)pdm09, influenza A(H1N1)pdm09; A(H3), influenza A(H3N2); RSV, respiratory syncytial virus.

For SARI cases, the average influenza and RSV hospitalization incidence rates were 1.7 and 1.9 per 1000 individuals, respectively. Notably, although the overall hospitalization impacts of influenza and RSV were similar to one another, the age‐stratified incidences were remarkably different (Table 4). Among children aged <6 months, the mean hospitalization rate owing to RSV was twofold to eightfold higher than that of influenza. This difference progressively decreased as the age of the patients increased, and the influenza hospitalization rate was higher than the RSV hospitalization rate in the ≥50 age group. For influenza‐related hospitalizations, influenza B had higher hospitalization rates than influenza A during this study period (0.9 and 0.3 per 1000 individuals, respectively). This difference partially reflected to persistent surge in the rate of children aged 5‐14 years.

**Table 4:** Incidence rates per 1000 person years of influenza‐ and RSV‐associated SARI by age groups, 2012‐2014

Year	<6 mo	6‐23 mo	2‐4 y	5‐14 y	15‐49 y	≥50 y
2012						
Influenza	5.6(2‐12.5)	8.9(4.1‐17)	3.9(1‐10)	0.8(0‐5.2)	0.2(0‐4.1)	2.3(0.3‐7.7)
A(H1)pdm09	0(0‐3.7)	0(0‐3.7)	0(0‐3.7)	0(0‐3.7)	0(0‐3.7)	0(0‐3.7)
A(H3)	1.7(0.14‐6.7)	4.3(1.26‐10.7)	2.4(0.38‐7.9)	0.6(0‐4.9)	0.2(0‐4)	1.6(0.14‐6.6)
Influenza B	2.1(0.3‐7.4)	2.9(0.6‐8.6)	1(0‐5.5)	0.1(0‐4)	0(0‐3.7)	0.4(0‐4.5)
RSV	44.0(32‐59)	24.6(15.8‐36.4)	5.4(1.9‐12.3)	0.5(0‐4.7)	0.2(0‐4)	1.6(0.1‐6.6)
2013						
Influenza	6.0(2.2‐13.1)	14.0(7.7‐23.5)	3.5(0.9‐9.6)	1.7(0.2‐6.8)	0.6(0‐4.9)	3.1(0.7‐9)
A(H1)pdm09	0(0‐3.7)	1.6(0.1‐6.5)	1.0(0‐5.5)	0.3(0‐4.2)	0.03(0‐3.7)	1.0(0‐5.6)
A(H3)	0(0‐3.7)	1.2(0.04‐5.9)	0.4(0‐4.4)	0.2(0‐4)	0(0‐3.7)	0.2(0‐4.1)
Influenza B	6(2.2‐13.1)	11.3(5.7‐20)	2.2(0.3‐7.5)	1.3(0.1‐6.1)	0.5(0‐4.7)	1.6(0.1‐6.6)
RSV	50.3(37.4‐66.2)	27.5(18.2‐39.9)	3.3(0.7‐9.2)	0.4(0‐4.4)	0.1(0‐3.9)	0.9(0‐5.4)
2014						
Influenza	17.1(10‐27.4)	15.7(8.9‐25.6)	3.3(0.8‐9.3)	1.8(0.2‐7)	0.4(0‐4.4)	6.2(2.3‐13.4)
A(H1)pdm09	1.1(0‐5.7)	0.8(0‐5.2)	0.2(0‐4.2)	0.1(0‐4)	0.01(0‐3.7)	0.2(0‐4.1)
A(H3)	3.3(0.75‐9.2)	4.7(1.46‐11.3)	0.8(0‐5.2)	0.5(0‐4.7)	0.2(0‐4)	3.2(0.69‐9)
Influenza B	12.8(6.7‐21.9)	9.7(4.6‐18)	2.3(0.3‐7.7)	1.2(0.1‐5.9)	0.1(0‐4)	2.9(0.6‐8.6)
RSV	64.4(49.6‐82.1)	38.0(26.9‐52.1)	6.4(2.4‐13.6)	0.2(0‐4.1)	0.1(0‐3.9)	1.2(0.1‐5.9)

SARI, severe acute respiratory illness; A(H1)pdm09, influenza A(H1N1)pdm09; A(H3), influenza A(H3N2); RSV, respiratory syncytial virus.

### 3.2 Health‐seeking behavior survey results

In total, 2655 respondents were enrolled in the health‐seeking behavior survey. The median age of the respondents was 10 years old (range: 1 month‐100 years of age), and the male‐to‐female ratio was 1:1.3. The results of the survey found that 822 ILI episodes had been experienced by the respondents over the previous 2 months, indicating that the favor to seek medical consultation during the dry season was 1.6‐fold that during the rainy season (Table 5). The facilities where these patients sought medical care were hospitals (28.8%), PHCs (25.2%), and private clinics (22.7%). Among the patients who visited PHCs, 67.6% sought medical consultation within 2 days from the onset, while 18.8% sought consultation when their symptoms worsened. The frequency of visiting PHCs was similar between residents of the city center and suburban areas (*P*=.57). The ILI frequency was significantly higher as it is to PHCs consultation levels we set (*P*=.09) (Table 5). Among the 267 SARI episodes reported by the survey respondents, 64.3% of cases sought care directly from hospitals. Of those cases, 74.4% visited hospitals within 2 days from the onset, while 14% visited when their symptoms worsened.

**Table 5:** Results of health‐seeking behavior survey in Baguio city, 2014

	No. of cases with ILI episode (%)	No. of cases with SARII episode (%)
Levels of PHCs’ consultation activities^a^		
Low	253 (29.7)	56 (6.6)
Medium	278 (31.0)	93 (10.4)
High	291 (32.1)	103 (11.4)
PHC Category		
Center	406 (32.2)	121 (9.6)
Suburb	416 (29.8)	131 (9.4)
Favor to consult during rainy season	370 (27.5)	n.a.
Favor to consult during dry season	282 (25.8)	n.a.

PHC, public health centers.

a Consultation activity was assessed with average number of ILI reported in 2012 and 2013.

ILI, influenza‐like illness.

### 3.3 Adjusted incidence rates for influenza and RSV

We adjusted our estimates of the influenza and RSV incidence rates based on the size of the residences of patients who did not visit PHCs, PHC consultation activity, and differences in health‐seeking behaviors during the rainy and dry seasons (Table 6). These adjusted influenza and RSV incidence rates were estimated as 4.8 and 2.3 per 1000 individuals, respectively. Notably, after these adjustments, the RSV incidence rate was remarkably high in children <6 months of age. The trends in the adjusted incidence rates of both influenza and RSV by age group are similar to the unadjusted incidence rates, which did not account for the group of infected individuals who did not seek medical attention; however, the adjusted incidence rates in adults >50 years were higher than the unadjusted incidence rates.

**Table 6:** Adjusted incidence rates per 1000 person years of influenza‐ and RSV‐associated ILI by age group in 2012‐2014

Year	<6 mo	6‐ 23 mo	2‐4 y	5‐14 y	15‐49 y	≥50 y
2012						
Influenza	5.1(1.7‐11.8)	14(7.6‐23.4)	12.7(6.7‐21.8)	4.8(1.5‐11.3)	0.3(0‐4.2)	0.4(0‐4.4)
A(H1)pdm09	0(0‐3.7)	0(0‐3.7)	0(0‐3.7)	0(0‐3.7)	0(0‐3.7)	0(0‐3.7)
A(H3)	3.7(1‐9.9)	9.4(4.4‐17.6)	10.3(5‐18.8)	4.1(1.1‐10.4)	0.2(0‐4.2)	0.3(0‐4.3)
Influenza B	1.4(0.1‐6.2)	4.6(1.4‐11.1)	2.4(0.4‐7.8)	0.7(0‐5)	0(0‐3.8)	0.1(0‐3.8)
RSV	34.3(23.8‐47.8)	37.1(26.1‐51)	11.8(6.1‐20.7)	1.7(0.2‐6.8)	0.3(0‐4.2)	0.4(0‐4.4)
2013						
Influenza	4.4(1.3‐10.8)	23.7(15.1‐35.3)	12.1(6.3‐21.1)	9.6(4.5‐17.8)	0.8(0‐5.3)	0.6(0‐4.8)
A(H1)pdm09	0(0‐3.7)	2.5(0.4‐8)	2.2(0.3‐7.5)	1.5(0.1‐6.5)	0(0‐3.8)	0.2(0‐4.2)
A(H3)	0(0‐3.7)	2.3(0.3‐7.7)	1.8(0.2‐6.9)	0.8(0‐5.3)	0(0‐3.7)	0.1(0‐3.8)
Influenza B	4.4(1.3‐10.8)	18.9(11.4‐29.5)	8.2(3.6‐16)	7.2(2.9‐14.7)	0.8(0‐5.2)	0.3(0‐4.2)
RSV	35.3(24.7‐49)	36.9(26‐50.8)	9.6(4.5‐17.9)	2(0.3‐7.3)	0.1(0‐3.9)	0.2(0‐4.1)
2014						
Influenza	12.7(6.7‐21.9)	18.4(11‐29)	8.9(4‐16.9)	9.2(4.3‐17.4)	0.4(0‐4.6)	0.9(0‐5.4)
A(H1)pdm09	0.5(0‐4.8)	1.9(0.2‐7.1)	0.4(0‐4.4)	0.4(0‐4.4)	0(0‐3.7)	0(0‐3.8)
A(H3)	6.4(2.4‐13.6)	6.6(2.6‐13.9)	3.1(0.7‐9)	2.2(0.3‐7.6)	0.2(0‐4.2)	0.6(0‐4.8)
Influenza B	5.8(2.1‐12.8)	9.9(4.7‐18.2)	5.4(1.8‐12.2)	6.6(2.6‐13.9)	0.2(0‐4.1)	0.3(0‐4.3)
RSV	27.4(18.1‐39.7)	40.8(29.3‐55.4)	13.2(7‐22.4)	0.8(0‐5.3)	0.1(0‐3.9)	0.2(0‐4.1)

ILI, influenza‐like illness; SARI, severe acute respiratory illness; A(H1)pdm09, influenza A(H1N1)pdm09; A(H3), influenza A(H3N2); RSV, respiratory syncytial virus.

## 4 Discussion

One global study estimated that there is a substantial influenza burden in children aged <5 years and in school‐aged children.^14^ We similarly found the highest hospitalization rate in children aged <2 years and the highest virus‐positivity rate in school‐aged children (5‐14 years old). These results reflect the identification of a large number of influenza B‐positive cases in these age groups. Higher hospitalization rates were observed for both influenza and RSV in adults aged >50 years than those aged 15‐49 years. As described elsewhere,^15^ the elderly comprise a large number of influenza hospitalizations and suffer severe outcomes. In agreement with a recent study that highlighted the role of RSV in adult hospitalizations,^16^ our data also suggest the need to monitor the RSV impact in this population and to consider the potential need for a control measure, such as vaccination.

Respiratory syncytial virus is a major etiological agent for both ILI and SARI cases among young children.^17^, ^18^ Although the overall RSV incidence rates of outpatients and inpatients in this study were comparable to those of influenza, we found that there was a higher incidence rate in young children for RSV than for influenza, which is in agreement with reports from previous studies.^7^, ^19^ Because the Philippines has a population structure with a high percentage of children, the burden of RSV is relatively high. There were very few fatalities among the study participants whose samples were determined to be positive for influenza or RSV. This is likely due in part to the early treatment that the study participants received, as three‐quarters of the subjects sought medical attention within 2 days. The small proportion of elderly individuals >65 years (3.5% of the total population) in our study, who typically contribute heavily to both influenza and RSV mortality,^20^, ^21^, ^22^ may have also contributed to the low number of fatalities in our study.

We estimated the incidence rates of influenza and RSV as threefold and 1.6‐fold, respectively, of the rates in the medically attended population. This is largely reflective of the size of the adolescent and adult population who did not seek medical care according to the health‐seeking behavior survey. These estimates are slightly lower than the rates estimated by a similar study conducted in Kenya.^7^ A surveillance system that targets the medically attended population can provide a solid range of incidence estimates for both individuals who receive medical attention and those who do not; however, the nature of such systems inherently includes the potential for underestimating overall incidence. Influenza episodes that do not receive medical attention lead to fewer direct economic losses than medically attended cases, but they impact indirect costs, such as productivity costs.^23^ Therefore, it is important to explore disease burden estimates in terms of both direct and indirect costs. Compared with the difference in the incidence rate between medically and non‐medically attended groups for influenza, less of a difference in incidence rates between these groups was observed for RSV in our study and the Kenyan study. The relatively young population that comprises the peak age group for RSV infection seeks medical care more frequently than the rest of the population.

We also found a difference in health‐seeking behavior between the rainy and dry seasons. Health‐seeking behavior has been widely used to calculate the function of the proportional range between medically attended and non‐medically attended groups.^7^, ^24^, ^25^ The results of a single cross‐sectional survey may better reflect recent episodes in respondents. That is, a question structure that considers the effect of a seasonal pattern may grasp behavioral attitudes more precisely than one that does not consider this factor.

There are some limitations in our study. First, owing to limited resources, we could not obtain samples from the ILI who visited facilities other than sampling days and from SARI cases whose onset was ≥5 days before sampling days, and this may have potentially lead to underestimates of the influenza and RSV incidence rates in both the medically attended and the non‐medically attended populations. Second, although we used the results of our health‐seeking behavior survey, which accounted for seasons as well as the levels of PHC consultation activity, to calculate a fraction of non‐medically attended individuals, these data were produced from a single survey. Although there was no significance difference in terms of the number of ILI and SARI cases between rainy and dry season, influenza A(H3) was predominantly circulated and both influenza B and RSV were less found. A variety of circulating viruses may affect the fraction of medically attended to non‐medically attended cases. We calculated the fraction of season by multiplying a ratio of proportions of medically attended between rainy and dry seasons to frequencies of seeking facilities in last 2 months prior to the interview conducted in dry season because we intended to minimize the effect of recall bias. A subsequent health‐seeking behavior survey in rainy season could estimate above fraction more precisely. Despite these limitations, this study provides incidence rates of both influenza and RSV among inpatients and outpatients that take into account the non‐medically attended population in the Philippines.

Respiratory syncytial virus and influenza pose a substantial disease burden in the city. To minimize this impact, particularly that of hospitalization, control measures, such as influenza vaccinations,^26^ should be considered.^27^ Additionally, the current facility‐based surveillance systems may underestimate the influenza incidence in infected individuals who do not seek medical attention. The implementation of either a community‐based approach or an enhanced surveillance system in combination with a community survey will allow a better understanding of the disease burdens of RSV and influenza in the Philippines.
